# Genetic Variations of *PTPN2* and *PTPN22:* Role in the Pathogenesis of Type 1 Diabetes and Crohn's Disease

**DOI:** 10.3389/fcimb.2015.00095

**Published:** 2015-12-24

**Authors:** Robert C. Sharp, Muna Abdulrahim, Ebraheem S. Naser, Saleh A. Naser

**Affiliations:** Burnett School of Biomedical Sciences, College of Medicine, University of Central FloridaOrlando, FL, USA

**Keywords:** PTPN22, PTPN2, T1D, Crohn's disease, genetics, immunity, autoimmunity, PTPs

## Abstract

Genome wide association studies have identified several genes that might be associated with increase susceptibility to Type 1 Diabetes (T1D) and Crohn's disease. Both Crohn's disease and T1D have a profound impact on the lives of patients and it is pivotal to investigate the genetic role in patients acquiring these diseases. Understanding the effect of single nucleotide polymorphisms (SNP's) in key genes in patients suffering from T1D and Crohn's disease is crucial to finding an effective treatment and generating novel therapeutic drugs. This review article is focused on the impact of SNP's in *PTPN2* (protein tyrosine phosphatase, non-receptor type 2) and *PTPN22* (protein tyrosine phosphatase non-receptor type 22) on the development of Crohn's disease and T1D. The *PTPN2* gene mutation in T1D patients play a direct role in the destruction of beta cells while in Crohn's disease patients, it modulates the innate immune responses. The *PTPN22* gene mutations also play a role in both diseases by modulating intracellular signaling. Examining the mechanism through which these genes increase the susceptibility to both diseases and gaining a better understanding of their structure and function is of vital importance to understand the etiology and pathogenesis of Type 1 Diabetes and Crohn's disease.

## Introduction

### Type 1 diabetes

**Figure 1:**
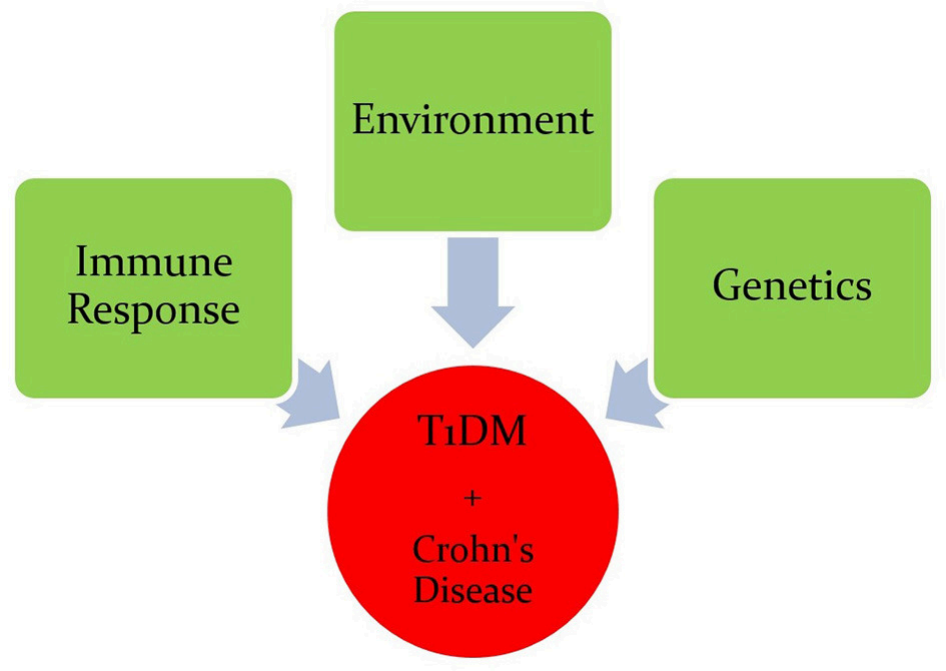
**Interplay between genetic predisposition, environmental factors, and the immune system in Crohn's disease and T1D**.

Type 1 Diabetes (T1D) is a chronic metabolic disorder that accounts for 5–10% of all diabetic cases. The incidence rate of T1D is steadily increasing and predicted to double in children under the age of 5 by 2020 (Todd, 2010). It has been suggested that T1D is a result of interplay between genetic predisposition, environmental factors, and reprograming of the immune system (Figure 1; Hober and Sauter, 2010). Regardless of the etiological factors of T1D, it is well-accepted that the destruction of pancreatic beta cells affects the level of insulin secretion leading to disease development. Destruction of pancreatic beta cells is mediated by an altered immune response due to genetic anomalies resulting in increase of pro-inflammatory cytokines and auto-reactive T and B lymphocytes. The impact of insulin deficiency includes hyperglycemia, ketoacidosis, kidney failure, heart disease, stroke, and possibly death (Naser et al., 2013).

Several environmental factors have been proposed to play a role in the development of T1D. This include diet, enteroviruses, and bacteria such as *Mycobacterium avium* subspecies *paratuberculosis* (MAP; van Belle et al., 2011). Vitamin D deficiency has been proposed to play a role in reprograming the immune system leading to pre-sensensitization to self-antigens (van Belle et al., 2011). The role of enteroviruses in T1D pathogenesis has been linked to upregulation of IFNα levels causing pancreatic beta cell damage (van Belle et al., 2011). Most recently, MAP has also been proposed. Naser et al. has demonstrated a high degree of homology (44% homology and 75% similarity over 16 amino acid sequences—potential epitope sites) between human pancreatic glutamic acid decarboxylase 65 kDa (GAD65) and MAP heat shock protein 65 kDa (Hsp65; Naser et al., 2013). The molecular mimicry between host GAD65, and the bacterial Hsp65 leads to reprograming of the immune system, and retargeting of host cells by pro-inflammatory cytokines and resulting in activation of autoantigen (Burn et al., 2011).

### Crohn's disease

**Figure 2:**
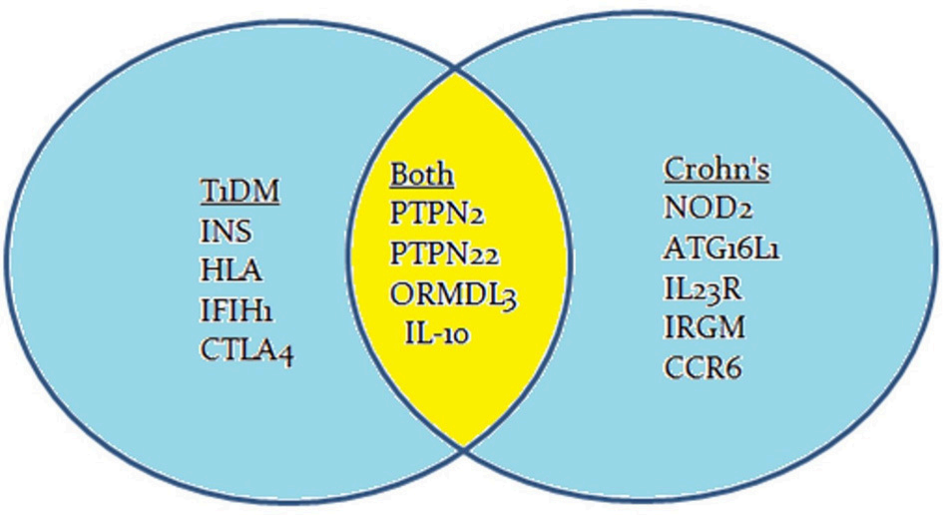
**Venn diagram of known susceptibility loci associated with both T1D and Crohn's disease**.

Crohn's disease is an inflammatory bowel disease (IBD) that is characterized by transmural inflammation of the intestinal wall, which may occur at different sites of the gastrointestinal tract (Scharl et al., 2009). IBD prevalence is rapidly increasing at an alarming rate. In a recent epidemiologic study in the State of Florida, United States, it was estimated that the prevalence of CD is 222 per 100,000 persons and the prevalence of UC is 307 per 100,000 persons in Florida. The prevalence of IBD was higher among people ages 30–80 years old, non-Hispanic Whites and females (Francois, 2006). The literature is enriched with reports suggesting that Crohn's disease, like T1D, is caused by multiple factors including genetic anomalies, environmental factors, and immune system malfunctions (Figure 1). The latter has significant impact on the pathophysiology of the disease including gut microbiota (Scharl et al., 2009). GWAS have shown that several candidate genes may cause an increase in the susceptibility to developing Crohn's disease (Barrett et al., 2008). These genes include: *NOD2, ATG16L1, IL23R, IRGM, CCR6, PTPN2*, and *PTPN22* (Figure 2; Barrett et al., 2008). Environmental factors that have been associated with Crohn's disease include pathogenic *Escherichia coli* strains, MAP, and others (Nazareth et al., 2015). Naser et al. has shown that MAP was found in the blood and breast milk of patients with Crohn's disease (Naser et al., 2000, 2004). Excessive secretion of pro-inflammatory cytokines and aberrant T cell differentiation have also exacerbated Crohn's disease, resulting in loss of tolerance, and intestinal dysbiosis (Sartor, 2006).

### Common genes associated with type 1 diabetes and Crohn's disease

Genome-wide association studies (GWAS) have identified many genes to be involved in the development of Crohn's disease or T1D or both. Most often, the mutation is due to a single nucleotide polymorphism (SNP) resulting in immune system impairment and ultimately increased susceptibility to disease. The susceptibility genes implicated to have a role in T1D pathogenesis include *INS, HLA, PTPN2, PTPN22, IL-10, IFIH1*, and *CTLA4* (Figure 2; van Belle et al., 2011; Naser et al., 2013). The potential role of some of these genetic mutations have been examined in previous studies, but the mechanisms by which these mutated genes play a role in T1D is still unclear and require further research.

GWAS have also suggested a possible association between other candidate genes and other diseases. Recently, two possible candidate genes that have been examined showed mutations in Crohn's disease and T1D. These genes are *PTPN2* (protein tyrosine phosphatase non-receptor type 2) and *PTPN22* (protein tyrosine phosphatase non-receptor type 22; Table 1; Scharl et al., 2012a). The *PTPN2* gene mutation in T1D patients plays a direct role in the destruction of beta cells, while in Crohn's disease patients, it modulates the innate immune responses (Barrett et al., 2008; Espino-Paisan et al., 2011). The *PTPN22* gene mutations also play a role in both diseases by modulating intracellular signaling (Barrett et al., 2008). *PTPN2* and *PTPN22* genes both encode for tyrosine phosphatases (PTPs) signaling molecules that modulate and regulate a variety of cellular processes such as cell growth, differentiation, mitotic cycle, oncogenic transformation, and survival (Estus et al., 2013). Studies have shown that PTPs in general are key regulators of signaling transduction. Most cells of the immune system show high expression of tyrosine phosphorylation and express more PTP genes than other tissues in the body. In fact, a distinct phenotype exists among PTP-knockout mice having deficient or hyperactive immune status with severe abnormalities in hematopoiesis. This suggests a crucial role of PTP in maintaining a balanced immune system (Chistiakova, 2010). Predisposing variants in these genes can potentially lead to a less efficient suppression of inflammatory response due to a reduced amount of negative regulation, which may contribute to diseases such as T1D and Crohn's disease.

**Table 1:** **Comparative analysis of the two protein tyrosine phosphatases associated with Type 1 Diabetes and Crohn's disease**.

	***PTPN2***	***PTPN22***
Location (Espino-Paisan et al., 2011)	Chromosome loci 18p11	Chromosome loci 1p13
Isoforms (Cerosaletti and Buckner, 2012)	Two major alternative splicing Endosplasmic reticulum and nucleus	Different: alternative splicing Cytoplasm
C-terminus domain (Cerosaletti and Buckner, 2012)	Variable	Long non-catalytic chain with several proline rich motif
N-terminus domain (Cerosaletti and Buckner, 2012)	Conserved catalytic	Phosphatase
SNP/rsID (T1D/CD; Barrett et al., 2008; Espino-Paisan et al., 2011)	Rs2542151-G variant allele	Rs2476601 R620W variant allele
Significant *P*-value for T1D Pathogenesis (Barrett et al., 2008; Smyth et al., 2008)	3.6 × 10^−15^	1.13 × 10^−88^
Significant *P*-value for CD Pathogenesis (Barrett et al., 2008; Smyth et al., 2008)	5.10 × 10^−17^	6.6 × 10^−6^

Legend: PTPN2, protein tyrosine phosphatase non-receptor type 2 gene; PTPN22, protein tyrosine phosphatase non-receptor type 22 gene; SNP, single nucleotide polymorphism; T1D, Type 1 Diabetes; CD, Crohn's disease.

## Genetic variations of *PTPN2* and *PTPN22*

### *PTPN2* role in type 1 diabetes and Crohn's disease

The *PTPN2* is located on chromosome 18 and is a member of the PTP family, which dephosphorylates receptor protein tyrosine residues and regulates many signaling pathways and processes. The protein has two major isoforms—one in the endoplasmic reticulum (48 kD) and the other in the nucleus (45 kD). PTPN2 is produced by alternative splicing and share a highly conserved PTP catalytic domain but different C-terminus (Cerosaletti and Buckner, 2012). *PTPN2* expression plays an important role in regulating signal transduction and it is of pivotal importance to the pathogenesis of many diseases such as T1D and Crohn's disease.

GWAS have showed that *PTPN2* is a T1D susceptibility gene and risk locus for Crohn's disease. In T1D, the association was obtained from chromosome region 18p11, which showed an associated risk variant at SNP rs2542151-G allele (Espino-Paisan et al., 2011). The involvement of the *PTPN2* gene in T1D is complex due to its ubiquitous expression which may play a role in beta cell apoptosis (Santin et al., 2011). This modulation occurs after exposure to type I (IFNα and IFNβ) and Type II interferon (IFNγ), which lead to beta cells loss in early T1D. Moreover, the study indicated that local IFN production interacts with *PTPN2* expression and induces a malfunctioning pro-apoptotic activity of Bim, a BH3-only protein (Santin et al., 2011). Bim is a member of the beta cell lymphoma two protein family (Bcl-2) that mediates apoptosis by activating Bax and Bak. This ultimately results in an increase of beta cell death via JNK activation and intrinsic apoptotic pathways (Santin et al., 2011). Many of T1D susceptibility genes are expressed in pancreatic beta cells indicating a possible self-destruction if these genes were mutated. The decrease of beta cell mass is driven in part by the release of cytokines and chemokines from immune cells. PTPN2 is a negative regulator of the JAK-STAT signaling pathway, which is activated downstream by IFN receptors. Studies have shown that *PTPN2* gene knockdown exacerbates type I and II IFN-induced beta cell death by inducing BAX translocation to the mitochondria after subsequent exposure to type I and II IFNs. This occurs because when *PTPN2* is mutated or knockdown, there is less of a negative regulation of apoptotic processes, thus, increasing the signaling of the destruction of cells. This reaction also releases cytochrome c and activates caspase three in human beta cells, which both induce apoptosis. Along with these reactions, there is an increase of Bim phosphorylation, which is regulated by JNK1 that also induces apoptosis of the β-cells. While examining this signaling cascade, *PTPN2* expression seems to be a putative factor in beta cell apoptosis (Santin et al., 2011). These findings imply that along with a genetic variation, the production of local IFN can lead to beta cell apoptosis as well as clarify the interplay between candidate genes and apoptosis in beta cells.

Recently, the role of PTPN2 in pancreatic endocrine function and insulin secretion was explored. In a study by Xi et al. the deficiency in *PTPN2* expression by knockout affected beta cell function in mice (Xi et al., 2015). The reduced insulin secretion wass associated with a decreased insulin content and glucose sensing, which showed that STAT3 could be a relevant target for the PTPN2 phosphatase regulation in the pancreas (Xi et al., 2015). PTPN2 regulates insulin signaling by inactivating its receptor through de-phosphorylation of the insulin receptor β-chain in conjunction with the PTP1B phosphatase. This regulates gluconeogenesis in the liver by attenuating STAT3 signaling, which decreases glucose levels (Fukushima et al., 2010; Cerosaletti and Buckner, 2012). A deficiency of PTPN2 expression will lead to a cytokine-induced beta cell apoptosis of the pancreatic cells after inducing the mitochondrial apoptotic pathway along with impacting glucose homeostasis/utilization (Fukushima et al., 2010; Cerosaletti and Buckner, 2012). With these two systems out of control, T1D could occur in patients who have a mutation in the *PTPN2* gene.

With the help of CD4+ helper T cells, CD8+ cytotoxic T cells are the primary mediators of beta cell destruction via secretory (perforin/granzyme) or Fas mediated pathways. Wiede et al. showed that a variant in *PTPN2* (rs1893217) in mice greatly increases T cell receptor signaling, which can lead to reduced self-antigen tolerance due to decreased negative regulation (Wiede et al., 2014). With this occurring, the response after self-antigen presentation could cause destruction of beta cells (Wiede et al., 2014). Moreover, the risk variant rs1893217 in the *PTPN2* gene is associated with a reduction in the receptor signaling of IL-2, which alters expression of FOXP3+ T regulatory cells (Tregs) in T1D patients (Cerosaletti and Buckner, 2012). Tregs are a group of T cells that modulate the immune system homeostasis by maintaining tolerance to self-antigens. They also prevent autoimmune diseases by acting as suppressors to the immune response. This dysregulation of FOXP3+ Tregs leads to both T cells and B cells being unregulated due to FOXP3+ Treg cells suppressing their activation (Cerosaletti and Buckner, 2012). With these altered FOXP3+ Tregs, over reactivity of both T cells and B cells could cause self-antigens to be recognized as foreign (Cerosaletti and Buckner, 2012). It explains how genetic variations in *PTPN2* could lead to the development of T1D due to the deregulation of Tregs homeostasis (Cerosaletti and Buckner, 2012). A SNP mutation (rs2542151-G) in the *PTPN2* gene is found not only in T1D, but in Crohn's disease as well (Espino-Paisan et al., 2011). In Crohn's disease, SNP variation in the *PTPN2* gene influences susceptibility to the disease and mechanism of pathogenesis.

It is a fact that epithelial barrier dysfunction coincides with immune response dysregulation in Crohn's disease. PTPN2 regulates intestinal epithelial barrier function and is activated by IFNγ which is up regulated by TNFα in intestinal epithelial cells (IEC; Osterman and Lichtenstein, 2007; McCole, 2012; Spalinger et al., 2015). IFNγ is an effector cytokine for Th-1 and potentially Th17-propagated immune responses (McCole, 2012; Spalinger et al., 2015). TNFα is a key effector of Cohn's disease in humans; therefore, anti-TNFα therapy is an effective treatment option for patients (Osterman and Lichtenstein, 2007). Scharl et al. showed that PTPN2 gets activated by IFNγ and in turn, it limits the pro-inflammatory cytokine-induced signaling and barrier defects. IFNγ plays a role in Crohn's pathogenesis and is noted to increase the permeability of intestinal epithelial barrier (Scharl et al., 2009). IFNγ is involved in tissue destruction and possibly, in reduction of barrier functions as a result of reconfigured tight junctions. PTPN2 usually protects the barrier by reducing its permeability and prevent induction of pore forming protein claudin-2. Claudin-2 is part of a family of proteins that regulates paracellular permeability and functions as sealer-like in tight junctions. Expressions or localization changes in claudins result in increased barrier permeability (Scharl et al., 2009). Recent study showed that *claudin-2* upregulation in Crohn's disease increased number of tight junction strand breakages (McCole, 2012). *PTPN2* expression plays a role in the regulation of inflammatory response, as loss of it leads to a severe IFNγ signaling cascade, leading to problems in the intestinal epithelial barrier function. PTPN2 has an important role in cytokine signaling of immune cells by inactivating STAT1 and STAT3; the loss of PTPN2 enhances STAT phosphorylation (Scharl et al., 2009). This evidence shows the importance in how a mutation altering function of the *PTPN2* gene could lead to deleterious effects and may explain the pathogenesis of associated diseases.

Loss of *PTPN2* expression is associated with increased expression and secretion of pro-inflammatory mediators in the intestinal epithelium (Scharl et al., 2009). As previously stated, there is an aberrant T cell differentiation and intestinal dysbiosis in Crohn's disease, which *PTPN2* seems to play a role in. It is very important to regulate T helper (Th) cell differentiation into effector T cell populations to maintain tolerance toward self-antigens and commensal bacteria of the intestine. There is a potential role of the PTPN2 protein in regulating differentiation of CD4+ Th cells into its subset population. A loss of the PTPN2 protein in T cells results in a disease promoting state. Loss of PTPN2 in T cell compartments leads to enhanced induction of Th1 and Th17 cells while having an impaired induction of regulatory T cells (Spalinger et al., 2015). In several mouse models as shown by Spalinger et al. increased inflammation occurred as a result of high numbers of Th1 and Th17 cells due to the loss of the PTPN2 protein function (Spalinger et al., 2015). Consequently, there is a disturbed immune response and altered intestinal microbiota in Crohn's disease that ultimately play a major role in its pathogenesis. Secretion of specific cytokines allows the Th cells to alter the immune response to commensal and pathogenic bacteria, and in doing so; it has a huge impact in the makeup of the intestinal microbiota (Spalinger et al., 2015). These studies further support the pivotal role of the *PTPN2* gene in Crohn's disease and the deleterious effects of a mutation within it.

*PTPN2* also plays a role in autophagosome formation in human intestinal cells. Autophagy is an essential process for maintaining cell homeostasis, survival, and modulating inflammation. Studies have shown that knockdown of *PTPN2* caused impaired autophagosome formation and dysfunctional autophagy resulting in response to TNFα and IFNγ. Moreover, silencing *PTPN2 in vitro* exacerbates intestinal epithelial barrier dysfunction when exposed to IFNγ (Scharl et al., 2012b). Impairment in this gene shows that the pathway that leads to the perpetual intestinal inflammation is associated with Crohn's disease patient. Loss of *PTPN2* expression can also lead to an increase in cytokine-induced mTOR phosphorylation, which leads to a decrease in autophagy. It was reported that *PTPN2* deficiency leads to a reduction of expression of autophagy genes that include: *beclin 1, ATG7, ATG5, ATG12* conjugates, and *ATG16L1* (Scharl and Rogler, 2012; Scharl et al., 2012b). Consequently, this leads to low amounts of autophagy proteins that create an abnormal autophagosome in the intestinal cells (McCole, 2012).

*PTPN2* expression is very important in immune regulation as can be noted with PTPN2 deficient mice that suffer severe inflammation and die swiftly after birth. A balance between inflammatory and regulatory T cells should be maintained for optimal tolerance and protection against pathogens (Spalinger et al., 2015). These observations show that the presence of the risk variants such as rs2542151-G within the *PTPN2* gene in Crohn's disease cause disease-associated characteristics (Espino-Paisan et al., 2011). The mutation in *PTPN2* could not only cause Crohn's disease but also T1D due to the presence of this mutation in both disease states. With this unregulated immune system due to the loss of PTPN2 function, cytokines that play a role in inflammation are substantially increased, and T cells/B cells begin to react to self-antigens. These changes will not only affect beta cells of the pancreas but should also affect the intestinal walls of these genetically susceptible patients, thus, they could cause either T1D or Crohn's disease.

### *PTPN22* role in type 1 diabetes and Crohn's disease

The *PTPN22* gene is located on chromosome 1p13 which is a member of the PTPs that negatively regulate T-cell activation (Cerosaletti and Buckner, 2012). The encoded protein is a lymphoid specific intracellular phosphatase that associates with the molecular adapter protein CBL. PTPN22 has alternatively spliced transcript variants encoding several distinct isoforms. It is located in the cytoplasm, and consists of an N-terminal phosphatase domain and a long non-catalytic C terminal with several proline rich motifs. PTPN22 dephosphorylates kinases Lck, Fyn, and ZAP70, which are all involved in T-cell signaling (Cerosaletti and Buckner, 2012). A SNP mutation (rs2476601) in *PTPN22* is associated with T1D and Crohn's disease (Barrett et al., 2008). Variants within these genes lead to the development of an abnormal immune response. *PTPN22* SNP mutation causes a single substitution of arginine for tryptophan in the encoded protein (R620W) leading to a decrease in T cell receptor and B cell receptor signaling (Menard et al., 2011). This may ultimately result in an unbalanced establishment of tolerance in both T cells and B cells (Menard et al., 2011).

In B cells, PTPN22 risk variant (R620W) prevents the removal of developing auto-reactive B cells. Menard et al. showed that new mature naive B cells from carriers of this variant had higher frequencies of auto-reactive clones as opposed to non-carriers (Menard et al., 2011). This demonstrates defective central and peripheral B cell tolerance checkpoints leading to the development of the previously mentioned auto-reactive B cells. To be noted, there are essentially two methods to removing autoreactive B cells. First, a central tolerance checkpoint is done to remove most of the developing B cells expressing polyreactive antibodies in the bone marrow (Wardemann et al., 2003). Second, a peripheral tolerance checkpoint is done in order to counter select autoreactive new B cells before entering compartments designed for mature naive B cell (Wardemann et al., 2003). This shows that a single risk allele would have a dominant effect of changing auto-reactive B cell counter-selection before onset of any autoimmunity. With regards to the decrease in T cell receptor signaling, it is possible that the T cell selection occurring in the thymus and T-regs in general, will be affected by this risk variant as well. Menard et al. also performed gene array experiments on mature naive B cells with the risk variant and found an upregulation of genes such as *CD40, TRAF1*, and *IRF5* (Menard et al., 2011). These genes encode proteins promoting B cell activation and are susceptibility genes of many deregulated immune diseases. They concluded that the association of the *PTPN22* gene with autoimmunity is due to impaired removal of auto-reactive B cells and the upregulation of the genes mentioned above (Menard et al., 2011).

In T cells, PTPN22 is directly involved in threshold setting for T cell receptor signaling (Vang et al., 2005). *PTPN22* SNP mutation results in a phosphatase with a higher catalytic activity and a more potent negative regulation of T cell lymphocyte activation (Vang et al., 2005). Recent studies on *PTPN22* knockout mice suggested that the increase risk of developing autoimmune diseases could occur through alterations of the periphery Treg cells while *PTPN22* knockout increases the thymic selection of Treg cells (Maine et al., 2012). Both Wu et al. and Zheng et al. also reported a “gain-of-function” model of Treg cell selection, where even though *PTPN22* knockout did have reduced TCR signaling, they did not have an impairment of their ability to negatively select autoreactive T cells in the thymus (Zheng and Kissler, 2013; Wu et al., 2014). Overall, *PTPN22* SNP mutation does not necessarily affect Treg cells, but could possibly affect other T cells once they leave out of the thymus or even have other effects on the immune system. Even though the role of *PTPN22* mutation is still debatable, both models (“gain-of-function”) and (“loss-of-function”) can still play a role in the development of T1D. The “gain-of-function” model does show that there is an increase in T cell activity, but this increase of mature T cell activation could be activated due to the loss of self-tolerance of peripheral T cells. With the “loss-of-function” model, it shows that if *PTPN22* is knocked out or mutated, then there is a loss of self-tolerance earlier on in the T cell life, which can then be activated by self-antigens.

*PTPN22* expression could potentially influence immuno-receptors, which could explain how it contributes to the development of diseases. Immuno-receptor signaling is governed by Src and Syk kinases, which are substrates of the PTPN22 protein (van Belle et al., 2011). A function of PTPN22 is to downregulate T-cell signaling by interacting with its negative regulatory kinase, C-terminal Src tyrosine kinase or Csk. A mutation of the *PTPN22* gene ends up encoding products with different Csk binding affinities (Ladner et al., 2005). The R620W substitution decreases the ability of the phosphatase to bind to the SH3 domain of Csk, thus, showing how *PTPN22* expression is associated with T cell signaling pathways. *PTPN22* C1858T SNP, which results in R620W substitution in the encoded protein, creates diabetes-specific autoimmunity. This can be used as a marker for disease progression by the noted appearance of autoantibodies. *PTPN22* SNP mutation seems to be associated with insulin-specific humoral autoimmunity (Hermann et al., 2006). Earlier studies reported that carriers of the risk allele are younger at the time of diagnosis of T1D, whereas *PTPN22* gene is a predictor of a more rapid progression of the disease (Hermann et al., 2006). Overall, *PTPN22* SNP contributes to failure in tolerance, which leads to the development of autoantibodies and subsequently, the disease (Cerosaletti and Buckner, 2012).

GWAS have also shown that SNP risk variant rs2476601 is present in Crohn's disease (Barrett et al., 2008). However, in Crohn's disease, it plays a complete opposite role to that in T1D (Barrett et al., 2008). The alteration in *PTPN22* expression levels and its dysfunction can have deleterious or beneficial effects depending on the mechanism involved. Normally, the intestinal immune system is usually tightly controlled by an existing balance of pro-inflammatory and anti-inflammatory cytokines. Patients suffering from IBD have a disturbed balance with more pro-inflammatory cytokines present. Crohn's patients have a reduced expression of *PTPN22* in intestinal tissues. Spalinger et al. showed that *PTPN22* expression regulates intracellular signaling as induced by IFNγ in human monocytes (Spalinger et al., 2013b). Studies have shown that knocking down the *PTPN22* gene alters the activation of inflammatory signal transducers and increases the secretion of Th17-related inflammatory mediators. By this mechanism, genetic variants may induce pathogenesis of Crohn's disease by prompting Th17 vs Th1 differentiation (Spalinger et al., 2013b). The decrease of *PTPN22* expression in Crohn's disease could be because TNFα and IL-1β both decrease its expression significantly and thus, play a role in its pathogenesis (Spalinger et al., 2013b). Spalinger et al. also found that the loss of PTPN22 protein function results in increased p38-MAPK but reduces STAT1 and STAT3 signaling (Spalinger et al., 2013b). This leads to increase levels of IL-6 and IL-17 secretion, and decrease expression and secretion of T-bet, ICAM-1, MCP-1, IL-2, IL-8, and IL-12p40. The reduced PTPN22 levels contribute to increased levels of IL-6 found in Crohn's disease. Also, p38 activation and IL-6 secretion by antigen presenting cells play a huge role in differentiation of CD4+ T cells into Th17 cells, which induces Crohn's disease pathogenesis (Spalinger et al., 2013b). The mechanism behind how *PTPN22* genetic variants are associated with Crohn's disease need to be further elucidated.

The function of the *PTPN22* gene in other signaling pathways is not very clear, but some studies have shown that it interferes with pathways induced by components of bacteria and it fine tunes signal cascade downstream of NOD2. NOD2 signaling is controlled by *PTPN22* expression and loss of it causes monocytes to be reactive toward bacterial components. This results in autophagy, which is increased in human and mouse cells by MDP (muramyl-dipeptide)-mediated mechanism (Spalinger et al., 2013a), further explaining association of this risk locus with IBD. PTPN22 plays an important role in cytokine secretion balance, which is crucial for activation and regulation of the immune system (Spalinger et al., 2013b). Mutations of PTPN22 not only will lead to cytokine imbalance, but it can also lead to T cells and B cells losing their ability to recognize self-antigens from foreign antigens. These imbalances can lead to the destruction of tissues (pancreatic for T1D patients and intestinal for Crohn's disease patients), which can become disastrous in the body.

## Conclusion

GWAS have played a key role in the discovery of important genes associated with diseases of unknown etiology. The protein tyrosine phosphatase genes, *PTPN2* and *PTPN22*, are of vital importance to both T1D and Crohn's disease. Several other genes such as *ORMDL3* and *IL-10* have also been associated with both T1D and Crohn's disease (Barrett et al., 2008; Qiu et al., 2014). This indicates that these categorized autoimmune diseases have common genetic predisposition that leads to the manifestation of their pathogenesis by different mechanisms or multiple pathways after exposure to environmental cues. Further research is needed to understand more about the actual cascade that occurs in the body and the mechanisms involved, as it is crucial to finding an effective treatment to deal with such diseases. A possible suggestion for future studies is to perform a large-scale GWAS investigation to estimate the incidence of SNP's in *PTPN2* and *PTPN22* in patients diagnosed with both T1D and Crohn's disease.

### Conflict of interest statement

The authors declare that the research was conducted in the absence of any commercial or financial relationships that could be construed as a potential conflict of interest.

## References

[B1] Barrett J. C. Hansoul S. Nicolae D. L. Cho J. H. Duerr R. H. Rioux J. D.. (2008). Genome-wide association defines more than 30 distinct susceptibility loci for Crohn's disease. Nat. Genet. 40, 955–962. 10.1038/ng.175 18587394 PMC2574810

[B2] Burn G. L. Svensson L. Sanchez-Blanco C. Saini M. Cope A. P. (2011). Why is PTPN22 a good candidate susceptibility gene for autoimmune disease? FEBS Lett. 585, 3689–3698. 10.1016/j.febslet.2011.04.032 21515266

[B3] Cerosaletti K. Buckner J. H. (2012). Protein tyrosine phosphatases and type 1 diabetes: genetic and functional implications of PTPN2 and PTPN22. Rev. Diabet. Stud. 9, 188–200. 10.1900/RDS.2012.9.188 23804260 PMC3740690

[B4] Chistiakova D. A. Chistiakova E. I. (2010). T-cell protein tyrosine phosphatase: a role in inflammation and autoimmunity. Int. J. Diabetes Mellit. 2, 114–118. 10.1016/j.ijdm.2010.05.012

[B5] Espino-Paisan L. de la Calle H. Fernández-Arquero M. Figueredo M. A. de la Concha E. G. Urcelay E.. (2011). A polymorphism in PTPN2 gene is associated with an earlier onset of type 1 diabetes. Immunogenetics 63, 255–258. 10.1007/s00251-010-0500-x 21246196

[B6] Estus J. L. Family Investigation of Nephropathy Diabetes Research Group Fardo, D. W. (2013). Combining genetic association study designs: a GWAS case study. Front. Genet. 4:186. 10.3389/fgene.2013.00186 24098305 PMC3784826

[B7] Francois M. R. (2006). Final Report of the Epidemiologic Study of Crohn's Disease and Ulcerative Colitis. Bureau of Epidemiology, Florida Department of Health.

[B8] Fukushima A. Loh K. Galic S. Fam B. Shields B. Wiede F.. (2010). T-cell protein tyrosine phosphatase attenuates STAT3 and insulin signaling in the liver to regulate gluconeogenesis. Diabetes 59, 1906–1914. 10.2337/db09-1365 20484139 PMC2911070

[B9] Hermann R. Lipponen K. Kiviniemi M. Kakko T. Veijola R. Simell O.. (2006). Lymphoid tyrosine phosphatase (LYP/PTPN22) Arg620Trp variant regulates insulin autoimmunity and progression to type 1 diabetes. Diabetologia 49, 1198–1208. 10.1007/s00125-006-0225-4 16614815

[B10] Hober D. Sauter P. (2010). Pathogenesis of type 1 diabetes mellitus: interplay between enterovirus and host. Nat. Rev. Endocrinol. 6, 279–289. 10.1038/nrendo.2010.27 20351698

[B11] Ladner M. B. Bottini N. Valdes A. M. Noble J. A. (2005). Association of the single nucleotide polymorphism C1858T of the PTPN22 gene with type 1 diabetes. Hum. Immunol. 66, 60–64. 10.1016/j.humimm.2004.09.016 15620463

[B12] Maine C. J. Hamilton-Williams E. E. Cheung J. Stanford S. M. Bottini N. Wicker L. S.. (2012). PTPN22 alters the development of regulatory T cells in the thymus. J. Immunol. 188, 5267–5275. 10.4049/jimmunol.1200150 22539785 PMC3358490

[B13] McCole D. F. (2012). Regulation of epithelial barrier function by the inflammatory bowel disease candidate gene, PTPN2. Ann. N. Y. Acad. Sci. 1257, 108–114. 10.1111/j.1749-6632.2012.06522.x 22671596 PMC5768569

[B14] Menard L. Saadoun D. Isnardi I. Ng Y. S. Meyers G. Massad C.. (2011). The PTPN22 allele encoding an R620W variant interferes with the removal of developing autoreactive B cells in humans. J. Clin. Invest. 121, 3635–3644. 10.1172/JCI45790 21804190 PMC3163953

[B15] Naser S. A. Ghobrial G. Romero C. Valentine J. F. (2004). Culture of *Mycobacterium avium* subspecies *paratuberculosis* from the blood of patients with Crohn's disease. Lancet 364, 1039–1044. 10.1016/S0140-6736(04)17058-X 15380962

[B16] Naser S. A. Schwartz D. Shafran I. (2000). Isolation of *Mycobacterium avium* subsp *paratuberculosis* from breast milk of Crohn's disease patients. Am. J. Gastroenterol. 95, 1094–1095. 10.1111/j.1572-0241.2000.01954.x 10763975

[B17] Naser S. A. Thanigachalam S. Dow C. T. Collins M. T. (2013). Exploring the role of *Mycobacterium avium* subspecies *paratuberculosis* in the pathogenesis of type 1 diabetes mellitus: a pilot study. Gut Pathog. 5:14. 10.1186/1757-4749-5-14 23759115 PMC3686596

[B18] Nazareth N. Magro F. Machado E. Ribeiro T. G. Martinho A. Rodrigues P.. (2015). Prevalence of *Mycobacterium avium* subsp. *paratuberculosis* and *Escherichia coli* in blood samples from patients with inflammatory bowel disease. Med. Microbiol. Immunol. 204, 681–692. 10.1007/s00430-015-0420-3 25994082

[B19] Osterman M. T. Lichtenstein G. R. (2007). Current and future anti-TNF therapy for inflammatory bowel disease. Curr. Treat. Options Gastroenterol. 10, 195–207. 10.1007/s11938-007-0013-3 17547858

[B20] Qiu Y. H. Deng F. Y. Li M. J. Lei S. F. (2014). Identification of novel risk genes associated with type 1 diabetes mellitus using a genome-wide gene-based association analysis. J. Diabetes Investig. 5, 649–656. 10.1111/jdi.12228 25422764 PMC4234227

[B21] Santin I. Moore F. Colli M. L. Gurzov E. N. Marselli L. Marchetti P.. (2011). PTPN2, a candidate gene for type 1 diabetes, modulates pancreatic beta-cell apoptosis via regulation of the BH3-only protein Bim. Diabetes 60, 3279–3288. 10.2337/db11-0758 21984578 PMC3219938

[B22] Sartor R. B. (2006). Mechanisms of disease: pathogenesis of Crohn's disease and ulcerative colitis. Nat. Clin. Pract. Gastroenterol. Hepatol. 3, 390–407. 10.1038/ncpgasthep0528 16819502

[B23] Scharl M. Mwinyi J. Fischbeck A. Leucht K. Eloranta J. J. Arikkat J.. (2012a). Crohn's disease-associated polymorphism within the PTPN2 gene affects muramyl-dipeptide-induced cytokine secretion and autophagy. Inflamm. Bowel Dis. 18, 900–912. 10.1002/ibd.21913 22021207

[B24] Scharl M. Paul G. Weber A. Jung B. C. Docherty M. J. Hausmann M.. (2009). Protection of epithelial barrier function by the Crohn's disease associated gene protein tyrosine phosphatase n2. Gastroenterology 137, 2030–2040. e2035. 10.1053/j.gastro.2009.07.078 19818778 PMC2855721

[B25] Scharl M. Rogler G. (2012). The role for protein tyrosine phosphatase nonreceptor type 2 in regulating autophagosome formation. Ann. N. Y. Acad. Sci. 1257, 93–102. 10.1111/j.1749-6632.2012.06578.x 22671594

[B26] Scharl M. Wojtal K. A. Becker H. M. Fischbeck A. Frei P. Arikkat J.. (2012b). Protein tyrosine phosphatase nonreceptor type 2 regulates autophagosome formation in human intestinal cells. Inflamm. Bowel Dis. 18, 1287–1302. 10.1002/ibd.21891 21987459

[B27] Smyth D. J. Plagnol V. Walker N. M. Cooper J. D. Downes K. Yang J. H.. (2008). Shared and distinct genetic variants in type 1 diabetes and celiac disease. N. Engl. J. Med. 359, 2767–2777. 10.1056/NEJMoa0807917 19073967 PMC2840835

[B28] Spalinger M. R. Kasper S. Chassard C. Raselli T. Frey-Wagner I. Gottier C.. (2015). PTPN2 controls differentiation of CD4(+) T cells and limits intestinal inflammation and intestinal dysbiosis. Mucosal Immunol. 8, 918–929. 10.1038/mi.2014.122 25492475

[B29] Spalinger M. R. Lang S. Vavricka S. R. Fried M. Rogler G. Scharl M. (2013a). Protein tyrosine phosphatase non-receptor type 22 modulates NOD2-induced cytokine release and autophagy. PLoS ONE 8:e72384. 10.1371/journal.pone.0072384 23991106 PMC3753240

[B30] Spalinger M. R. Lang S. Weber A. Frei P. Fried M. Rogler G.. (2013b). Loss of protein tyrosine phosphatase nonreceptor type 22 regulates interferon-gamma-induced signaling in human monocytes. Gastroenterology 144, 978–988. e910. 10.1053/j.gastro.2013.01.048 23380085

[B31] Todd J. A. (2010). Etiology of type 1 diabetes. Immunity 32, 457–467. 10.1016/j.immuni.2010.04.001 20412756

[B32] van Belle T. L. Coppieters K. T. von Herrath M. G. (2011). Type 1 diabetes: etiology, immunology, and therapeutic strategies. Physiol. Rev. 91, 79–118. 10.1152/physrev.00003.2010 21248163

[B33] Vang T. Congia M. Macis M. D. Musumeci L. Orrú V. Zavattari P.. (2005). Autoimmune-associated lymphoid tyrosine phosphatase is a gain-of-function variant. Nat. Genet. 37, 1317–1319. 10.1038/ng1673 16273109

[B34] Wardemann H. Yurasov S. Schaefer A. Young J. W. Meffre E. Nussenzweig M. C. (2003). Predominant autoantibody production by early human B cell precursors. Science 301, 1374–1377. 10.1126/science.1086907 12920303

[B35] Wiede F. Ziegler A. Zehn D. Tiganis T. (2014). PTPN2 restrains CD8(+) T cell responses after antigen cross-presentation for the maintenance of peripheral tolerance in mice. J. Autoimmun. 53, 105–114. 10.1016/j.jaut.2014.05.008 24997008

[B36] Wu D. J. Zhou W. Enouz S. Orru V. Stanford S. M. Maine C. J. (2014). Autoimmunity-associated LYP-W620 does not impair thymic negative selection of autoreactive T cells. PLoS ONE 9:e86677 10.1371/journal.pone.0086677 24498279 PMC3911918

[B37] Xi Y. Liu S. Bettaieb A. Matsuo K. Matsuo I. Hosein E.. (2015). Pancreatic T cell protein-tyrosine phosphatase deficiency affects beta cell function in mice. Diabetologia 58, 122–131. 10.1007/s00125-014-3413-7 25338551 PMC4258175

[B38] Zheng P. Kissler S. (2013). PTPN22 silencing in the NOD model indicates the type 1 diabetes-associated allele is not a loss-of-function variant. Diabetes 62, 896–904. 10.2337/db12-0929 23193190 PMC3581188

